# Anticonvulsant Essential Oils and Their Relationship with Oxidative Stress in Epilepsy

**DOI:** 10.3390/biom9120835

**Published:** 2019-12-06

**Authors:** Diogo Vilar da Fonsêca, Carlos da Silva Maia Bezerra Filho, Tamires Cardoso Lima, Reinaldo Nóbrega de Almeida, Damião Pergentino de Sousa

**Affiliations:** 1 College of Medicine, Federal University of the Vale do São Francisco, Paulo Afonso, BA, CEP 48607-190, Brazil; divilar@hotmail.com; 2 Department of Pharmaceutical Sciences, Universidade Federal da Paraíba, João Pessoa, PB, CEP 58051-970, Brazil; carlosmaia1996@gmail.com; 3 Department of Pharmacy, Federal University of Sergipe, São Cristóvão, SE, CEP 49100-000, Brazil; tamires.cl87@gmail.com; 4 Department of Physiology and Pathology, Universidade Federal da Paraíba, João Pessoa, PB, CEP 58051-970, Brazil; reinaldoan@uol.com.br

**Keywords:** terpene, phenylpropanoid, natural products, seizures, pentylenetetrazole, electroshock, antioxidants, phytochemicals, secondary metabolites, bioactive

## Abstract

Epilepsy is a most disabling neurological disorder affecting all age groups. Among the various mechanisms that may result in epilepsy, neuronal hyperexcitability and oxidative injury produced by an excessive formation of free radicals may play a role in the development of this pathology. Therefore, new treatment approaches are needed to address resistant conditions that do not respond fully to current antiepileptic drugs. This paper reviews studies on the anticonvulsant activities of essential oils and their chemical constituents. Data from studies published from January 2011 to December 2018 was selected from the PubMed database for examination. The bioactivity of 19 essential oils and 16 constituents is described. Apiaceae and Lamiaceae were the most promising botanical families due to the largest number of reports about plant species from these families that produce anticonvulsant essential oils. Among the evaluated compounds, β-caryophyllene, borneol, eugenol and nerolidol were the constituents that presented antioxidant properties related to anticonvulsant action. These data show the potential of these natural products as health promoting agents and use against various types of seizure disorders. Their properties on oxidative stress may contribute to the control of this neurological condition. However, further studies on the toxicological profile and mechanism of action of essential oils are needed.

## 1. Introduction

Epilepsy, one of the most prevalent chronic neurological disorders globally, is characterized by recurrent, unpredictable, and typically unprovoked seizures. Although subjects from any age group can develop epilepsy, the fastest-growing population segments for new cases are young children and older adults. Roughly two-thirds of initial seizures occur in young children under the age of 2, and in the elderly (over 75 years of age), the prevalence is 3% [1,2]. According to the WHO, at least 50 million people worldwide are affected by epilepsy [3].

**Figure 1:**
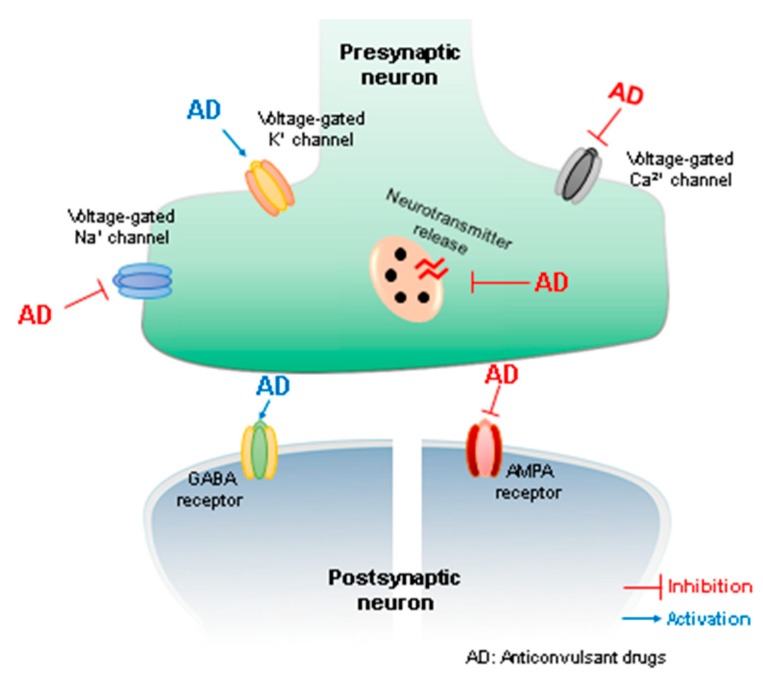
Main mechanisms of action of anticonvulsant drugs.

Epileptic seizures impact the patient’s quality of life, and usually include hospitalization with risks of cognitive and motor impairment, psychological distress, progressive memory loss, social stigmatization, and isolation [4]. Although a large number of anticonvulsant drugs are available for treatment of epilepsy, and new drugs have brought more treatment options, around 30% of the epilepsy cases remain pharmaco-resistant, not responding to anti-seizure drug therapy or patients discontinue treatment due to its serious side effects [5]. Figure 1 summarizes the main mechanisms of action of anticonvulsant drugs.

Many species of aromatic plants contain biologically active compounds with the potential to act in various chronic conditions, such as anxiety, depression and headaches. Some act in the Central Nervous System (CNS), and are used in folk medicine to treat epilepsy because of its anticonvulsive activity [6,7]. Various studies in essential oils (EOs) and particularly their chemical constituents have been noted in the scientific community, and encourage further study of their properties. Such secondary metabolites have brought great potential for new anticonvulsant drug development, with minimal untoward harmfulside effects, and thus novel treatment strategies for patients that do not respond well to conventional therapies [6].

### Relationship between Epilepsy and Oxidative Stress

The oxidative stress is a metabolic occurrence in which there is a disturbance in the balance between pro-oxidant and antioxidant species [8]. Pro-oxidant agents are mainly represented by reactive oxygenated (ROS) and nitrogenous species (RNS) [9]. They are normally neutralized by an antioxidant defense system composed of enzymes such as catalase (CAT), superoxide dismutase (SOD), and glutathione peroxidase (GPx), and of non-enzymatic compounds such as vitamins A, C, and E, which help maintain homeostasis. However, in pathological situations, exacerbated production of ROS and/or RNS may occurresulting in oxidative stress [10]. 

The brain is an organ sensitive to oxidative stress due to some of its features such as high oxygen demand, large numbers of mitochondria, low repair capacity, and high polyunsaturated fatty acid concentrations [11]. In addition, the brain maintains only low levels of antioxidants which are mainly in the hippocampus [12]. This event increases the predisposition towards oxidative stress. Several studies reveal that cases of seizure induce the production of ROS and RNS [13,14], promoting oxidative stress that results in modulation of nucleic acid, protein and lipid peroxidation functions, resulting in cell damage [15]. 

Studies show that epilepsy carriers have elevated serum malondialdehyde (MDA) levels in most cases due to increased membrane lipid peroxidation [16], which causes changes in neurotransmitter release and uptake and ion channels expression resulting in neuronal hyperexcitability [17]. In addition, the production of ROS promotes increased cytoplasmic Ca^2+^ concentrations, enhancing neuronal hyperexcitability, directly influencing GABA_A_ receptor action, altering neuronal membrane potential, and promoting gene transcription modifications and protein synthesis, inducing changes in neuronal physiological functions. These changes may result in neuronal death by necrosis or apoptosis, being one of the most relevant factors that may lead to the development of epileptic condition [18,19].

Mitochondria directly influence neuronal excitability, adenosine triphosphate (ATP) production, fatty acid oxidation, apoptosis control, neurotransmitter biosynthesis, and regulation of intracellular calcium homeostasis. The production of reactive oxygen species occurs mainly in mitochondria, which are vulnerable to oxidative damage. Thus, oxidative stress promotes mitochondrial dysfunction [20,21,22]. This dysfunction can trigger epileptic seizures via reduced ATP production, altering the Na^+^/K^+^ ATPase activity present in the cell membrane, responsible for maintaining the membrane potential, thus reducing its activity and increasing neuronal excitability [23,24].

In addition, oxidative stress and mitochondrial dysfunction contribute to glutamate-induced excitotoxicity and later neuronal apoptosis [25], and death of hippocampal neurons is quite recurrent in cases of epilepsy, contributing to cognitive dysfunction [26]. Thus, substances that have antioxidant activity can help treat epilepsy by reducing cerebral oxidative stress. Studies indicate that treatment with antioxidant agents promotes neuroprotection, reduces neuronal apoptosis, as well as improved cognitive dysfunction in cases of epilepsy [27,28].

Therefore, this review discusses on current studies of EOs with anticonvulsant activity and their relationship to oxidative stress. Further, the chemical structures of their bioactive constituents, their mechanisms of action, and the experimental models used are also presented and discussed.

## 2. Anticonvulsant Essential Oils

### 2.1. Bunium persicum *(Boiss). B. Fedtsch.*

*Bunium persicum* is a grassy plant belonging to the family Apiaceae, found mainly in southeastern Iran [29]. The seeds of this plant are widely used in traditional Iranian medicine for their spasmolytic, antiepileptic, and carminative effect [30]. Analysis of *B. persicum* essential oil (BPEO) seeds using gas chromatography coupled to mass spectrometry (GC/MS) allowed identification of 97.2% of the constituents; the main compounds were γ-terpinene (46.1%), cuminaldehyde (15.5%), *p*-cymene (6.7%), and limonene (5.9%) [31]. According to the literature, BPEO presents anti-toxoplasma [31], antioxidant [32], antinociceptive and anti-inflammatory activity [33]. Nickavar et al. (2014) evaluated the antioxidant and antilipid peroxidation activity of BPEO using 2,2-diphenyl-1-picrylhydrazyl (DPPH) radical scavenging and linoleic acid/β-carotene bleaching assays, respectively. According to the results, the essential oil presented good radical scavenging [half maximal inhibitory concentration (IC_50_) = 4.47 mg/mL], and inhibited lipid peroxidation (IC_50_ = 0.22 mg/mL) [34] Also, BPEO exhibited antioxidant activity using the Ferric Reducing Antioxidant Power (FRAP) assay (IC_50_ = 248.56 ± 1.09 µM Fe^2+^/g) [35]. 

The results obtained during evaluation of anticonvulsant activity for BPEO were quite promising (Table 1). In the pentylenetetrazol (PTZ)-induced seizure test, BPEO prolonged the onset time to clonic and tonic seizures, at (ED_50_) = 0.97 mL/kg. The potency of BPEO effects was also verified in the maximal electroshock induced convulsions test in which significant prevention of tonic convulsion at (ED_50_ = 0.75 mL/kg) was observed. Signs of neurotoxicity analyzed in the Rota-rod test appeared at the dose of 1.25 mg/kg which decreased the time of permanence of the animal on the rotatory rod. However, mortality was not observed until a dose of 2.5 mL/kg, i.p. [36].

### 2.2. Calamintha officinalis *Moench*

The shrub *Calamintha officinalis* Moench (Lamiaceae), is widely found throughout the Mediterranean [37]. Since ancient times, *C. officinalis* has been used for its medicinal antidiarrheal, expectorant and antibacterial properties. In Morocco, the plant is used to treat hypertension, cardiovascular diseases, and diabetes [38]. Few studies have reported on the biological activities of *C. officinalis* leaves and essential oil (COEO) although antimicrobial, sedative, and hypothermic effects have been described [39,40]. In COEO analysis using GC/MS, sixty-four compounds were identified, constituting 99.7% of the total oil. The main component is the oxygenated monoterpene carvone (38.7%), followed by neo-dihydrocarveol (9.9%), dihydrocarveol acetate (7.6%), dihydrocarveol (6.9%), 1,8-cineole (6.4%), *cis*-carvyl acetate (6.1%), and pulegone (4.1%). In PTZ-induced seizure tests, COEO (50 and 100 mg/kg, i.p.) increased latency times, reduced the number of animal seizures, and decreased seizure duration. Carvone, a major compound, presents a demonstrable anticonvulsive effect; however other constituents may have synergistic activities that explain COEO’s depressant activity [41].

### 2.3. Cinnamosma madagascariensis *Danguy*

The genus *Cinnamosma*, belonging to Canellaceae family, is endemic in Madagascar, and *C. madagascariensis* is widely found in both southeastern and southern Madagascar [42]. Popularly known as “hazontromba”, *C. madagascariensis* is used to treat coughs and strengthen the immune system. Burning its leaves is used in rituals to scare away evil spirits and to quell brain disorders such as dementia and epilepsy [43]. The most abundant components *C. madagascariensis* leaves’ essential oil (CMEO), are monoterpene hydrocarbons (40.0%), oxygenated monoterpenes (35.7%), linalool (30.1%), limonene (12.0%), myrcene (8.9%), and α-pinene 140 (8.4%) as [44]. In PTZ testing in rats, CMEO, at a dose of 0.8 mL/kg, protected against convulsions, and at a dose of 0.4 mL/kg increased latency and reduced both seizure frequency and severity [45]. These promising CMEO results occur in function of synergism between the constituents, since linalool, limonene, and myrcene already present anticonvulsive activities well described in the literature [46,47]. 

### 2.4. Citrus aurantium *L. var.* Amara

*Citrus aurantium* is popularly known as “bitter orange”. The flowers of *C. aurantium* are used to treat various neurological disorders such as insomnia, epilepsy, and hysteria [48]. Some pharmacological activities have already been described for *C. aurantium* essential oil (CAEO) such as an anti-inflammatory agent [49], an anxiolytic [50], as a larvicide [51] and antioxidant [52]. 

Intraperitoneal administration CAEO from fresh blossoms at doses of 20 and 40 mg/kg increased the clonic convulsion threshold and protected against tonic convulsion induced by intravenous PTZ. The combination of an ineffective dose of CAEO (10 mg/kg) together with an ineffective dose of diazepam (0.025 mg/kg) provided an additive effect for protection against PTZ-induced seizures. The observed activity may be related to benzodiazepine receptor activation. The main constituents of CAEO from fresh blossoms (which may be responsible for the pharmacological activity) are linalool (28.5%), linalyl acetate (19.6%), nerolidol (9.1%), farnesol (9.1%), α-terpineol (4.9%), and limonene (4.6%) [53].

### 2.5. Dennettia tripetala *G. Baker*

*Dennettia tripetala* is a medium-sized tropical plant belonging to the Annonaceae family and is used as a flavoring agent. When added to the diet of pregnant and postpartum women it helps uterine contraction and involution [54]. It is widely cultivated in the rainforests of West Africa, including southeast and south-west Nigeria [55]. The fruits are eaten raw in different forms, while the leaves are used as condiments in local dishes [56]. Essential oil from the fruits of *D. tripetala* present antimicrobial, anti-inflammatory, antinociceptive, and antioxidant activity. In these studies with DPPH, lipid peroxidation, and nitric oxide radical, the essential oil was more effective than ascorbic acid in elimination of free radicals. This activity should involve monoterpenes which are part of their chemical composition [57]. Oyemitan et al. [58] extracted essential oil from the seeds of *D. tripetala* (DTEO) and evaluated its anticonvulsant activity at doses of 25, 50, and 100 mg/kg (i.p.). These doses respectively protected the animals from PTZ-induced seizures by 20%, 40%, and 100%. This effect was blocked by flumazenil, suggesting that DTEO exerts its effect via interaction with the GABAergic system. In the strychnine-induced seizure test, convulsions were respectively protected at doses of 100 and 200 mg/kg, by 60% and 100%. At these doses, mortality was also reduced. These results can be attributed to 1-nitro-2-phenylethane, the major compound which in the same study also demonstrated a potent anticonvulsant effect.

### 2.6. Elettaria cardamomum *L. Maton*

Cardamom, *Elettaria cardamomum*, is an aromatic plant of the Zingiberaceae family grown in some Asian countries [59]. In India, the dried seeds are used as flavoring agents in teas, cakes, and coffee [60], and also in traditional medicine to treat diarrhea, colic, constipation, asthma, and epilepsy [61]. According to Abu-Taweel, cardamom added to the rations of pregnant mice improved both memory and learning, and even brought perinatal benefits since the compounds can be transported via the placenta and/or during lactation [62]. Also, anxiolytic effects have been reported in the literature for cardamom [63]. Analysis of the chemical composition of *E. cardamomum* essential oil (ECEO) by GC/MS identified 93.5% of the constituents; 1,8-cineole (45.6%), α-terpinyl acetate (33.7%), terpinen-4-ol (2.4%), and myrcene (2.2%) [64].

Since many terpenes have anticonvulsive effect, interest arose in assessing this activity in ECEO. In the PTZ-induced seizure test, ECEO at a dose of 1 mL/kg (i.p) delayed the onset of clonic seizure and increased tonic seizure latency at all doses tested (0.25, 0.50, 0.75, 1.00 mL/kg, i.p.). In the maximal electroshock seizure (MES) test, ECEO decreased the percentage of tonic extension of the posterior limb caused by electrical stimulus. Possible neurotoxic activities of ECEO were also observed at a dose of 0.75 mL/kg [64].

### 2.7. Gardenia lucida *Roxb.*

*Gardenia lucida* is a plant found in India and belongs to the Rubiaceae family. The leaf buds secrete a gum-like resin that resembles yellow tears with a strong odor and spicy taste [65]. In traditional Indian medicine, this gum-resin is used as an antispasmodic, antimicrobial, carminative, and anthelmintic [66]. Some studies suggest neuropharmacological activities for *G. lucida* essential oil from this gum-resin (GLEO). At a lethal dose it caused the death of 50% of a group of test animals (LD_50_) greater than 5 mL/kg, yet without showing toxicological signs [67]. GS/MS analysis revealed the presence of 18 constituents, of which α-pinene (45%) and spathulenol (31%) were the main components. Doses of 100 and 300 mg/kg, i.p. of GLEO presented CNS depressant effect, which was potentiated by the administration of a barbiturate. The anticonvulsant activity was initially evaluated using the PTZ test in which at 300 mg/kg, seizure delay (395.6%) was observed, seizure frequency decreased by 257% when compared to the control group, and the reduction in the mortality rate was 83%. In the electroshock test, there was an increase in tonic flexion (100 and 300 mg/kg, i.p.), and reductions in tonic extension (30, 100, and 300 mg/kg, i.p.) [67]. 

### 2.8. Pimpinella anisum *L.*

*Pimpinella anisum* is a member of the family Umbelliferae (Apiaceae) and is used for cooking and in Iranian medicine as a carminative, diuretic and for treatment of epilepsy and melancholia [68]. *P. anisum* essential oil (PAEO), also called anise oil, presents *trans*-anethole (89.1%), estragol (3.6%), linalool (1.1%), α-terpineol and *cis*-anethole (0.2%) as its main constituents [69].

Anise oil’s medicinal properties have already been proven and include treatment of non-fatty liver diseases [70]; antidepressant [71], antifungal [72] and antioxidant properties capable of eliminating up to 48% of the free radicals generated in DPPH assay using 48.5 mg/mL [73,74]. 

In a study by Karimzadeh et al. (2012) [75], it was observed that concentrations at 1 and 2 mL/kg of PAEO did not alter seizure latency after administration of PTZ. However, a 3 mL/kg dose significantly prolonged the time to onset of seizure. During electroencephalographic recording, PAEO in the three doses tested (1, 2, and 3 mL/kg) promoted significant decreases in the frequency, amplitude, and duration of the epileptiform burst discharges induced by PTZ injection. In the same study, the neuroprotective effect of PAEO in epileptic rats was evidenced due to a significant decrease in the production of dark neurons, possibly through synaptic plasticity inhibition.

### 2.9. Piper guineense *Schum &Thonn*

This plant of the family Piperaceae is widely consumed as spice in West Africa. It is used in popular culture to treat infertility in women, rheumatism, intestinal disorders, bronchitis, and febrile seizure [76,77]. Study results relate analgesic, anti-parasitic [78], hepatoprotective [79], molluscicide [80], sedative and anxiolytic activity [81]. In the attempt to identify the chemical constituents responsible for these effects, the essential oil from the fresh fruits of *P. guineense* was analyzed and sesquiterpenes (64.4%) were predominant, while monoterpenes represented only 21.3%, with the presence of β-sesquiphellandrene (20.9%), linalool (6.1%), limonene (5.8%), β-bisabolene (5.4%) and α-pinene (5.3%) [82]. *Piper guineese* essential oil (PGEO) presented antioxidant activity in tests of DPPH, nitric oxide radical scavenging assays and Fe^2+^ chelation assays, obtaining EC_50_ results of 414.59 mL/L, 161.92 mL/L, and 130.21 mL/L, respectively [83].

PGEO at doses of 100 and 200 mg/kg (i.p.) respectively protected animals by 40% and 100% against PTZ-induced seizures and also decreased the mortality test rate. In the same study, it was observed that PGEO demonstrated hypothermic, sedative, muscle relaxant, and antipsychotic activity which explains its ethno medicinal use [82]. Described in the literature, linalool presents anticonvulsive activity and may contribute synergistically or add to the activity of other EOPG phytochemicals and the oil’s resulting pharmacological effects [84,85].

### 2.10. Smyrnium cordifolium *Boiss.*

The plant *Smyrnium cordifolium* is used in Iranian medicine to treat anxiety, insomnia, and internal organ edemas (mainly of the liver and kidneys) [86]. Some of the pharmacological properties of *S. cordifolium* extract have already been described, including hypnotic, antimicrobial, and antioxidant effects [87,88,89]. *Smyrnium cordifolium* essential oil (SCEO) consists mainly of curzerene (65.26%), δ-cadinene (14.39%), and γ-elemene (5.15%) [90]. In the PTZ-induced seizure test, SCEO presented an ED_50_ value of 223 ± 15 mg/kg. The anticonvulsant effect was suppressed by previous administration of flumazenil and naloxone, suggesting GABAergic and opioid system participation [90].

### 2.11. Thymus vulgaris *L.*

*Thymus vulgaris* L. (Lamiaceae) is an aromatic plant whose essential oil already has various pharmacological activities described such as antimicrobial [91], anthelmintic [92] and anti-inflammatory [93]. The GC-MS analysis of *Thymus vulgaris* essential oil (TVEO) revealed the presence of more than 50 metabolites. The main compounds were thymol (34.78%), *p*-cymene (14:18%), carvacrol (6:16), β-caryophyllene (5:46%), linalool (3.83%), terpinen-4-ol (2.56%), caryophyllene oxide (2.31%), and borneol (2.22%) [94]. TVEO (300 mg / kg, ip) reduced the number of seizures induced by MES when administered 15 and 30 min before the test (50 and 62.5%, respectively), but at times 45 and 60 min it was not longer effective, probably due to metabolization and elimination of the active metabolites. In the same study, it was observed that administration of the isolated components, borneol, thymol and eugenol at a dose of 300 mg/kg, i.p. protected against seizures induced by MES, different from linalool which had no good result. Thus, these active components are believed to act synergistically to provide the anticonvulsant effect of TVEO [94].

### 2.12. Zataria multiflora *Boiss.*

*Zataria multiflora* is an aromatic plant belonging to the family Lamiaceae, which grows in the hot and mountainous regions of Iran, Pakistan, and Afghanistan [95]. The essential oil of *Z. multiflora* (ZMEO) is rich in oxygenated phenolic monoterpenes; mainly carvacrol, linalool, *trans*-caryophyllene and carvacrol methyl ether [96]. Certain scientific reports demonstrate its antileishmanial [97] and antibacterial activity [98] of ZMEO. Majlessi et al. [99] demonstrated that ZMEO is a potential therapeutic agent in alleviating the cognitive symptoms of Alzheimer’s disease. Kavoosi et al. [100] investigated the antioxidant capacity of ZMEO, and showed that ZMEO at IC_50_ = 4.2 μg/mL in a reactive nitrogen scavenging assay that it significantly reduces NO and H_2_O_2_ production, reducing NO synthase and NADH oxidase activity in macrophages. Corroborating these results, Karimian et al. [101] compared the antioxidant activity of ZMEO and vitamin C using the NO scavenging test, where they respectively presented values of 38 μg and 46.5 μg; indicating the remarkable antioxidant capacity of this oil and its potential for use in oxidative damage therapy.

Mandegary et al. [102] evaluated the anticonvulsant activity of ZMEO and observed that at doses of 0.2, 0.25, and 0.35 mL/kg there was an increase in clonic seizure onset time, and tonic convulsions induced by PTZ were prevented using the same doses in mice. However, ZMEO was not effective in the MES test. When assessing the neurotoxic potential in the Rota-rod test, ZMEO at a dose of 0.6 mL/kg caused changes in motor coordination. The LD_50_ value of ZMEO was determined to be 1.30 (1.0-1.5) mL/kg.

### 2.13. Zhumeria majdae *Rech.*

*Zhumeria majdae* is a member of the Lamiaceae family, and found in southeastern Iran. This common plant presents a strong odor and its leaves are used as an antiseptic to treat digestive disorders and dysmenorrhea [103]. Although it is widely used in folk medicine, there are few studies on this plant in the literature. Studies have revealed the antifungal [104], anti-bacterial [105], antinociceptive, anti-inflammatory [106], antileishmanial [107] and antioxidant [108] activity of *Z. majdae* essential oil and extract. Analysis of *Z. majdae* essential oil (ZHMEO) by GC and GC-MS revealed the existence of seventy constituents representing 99.2% of the oil. Since these terpenoids have anticonvulsive activity, the activity of ZMEO was evaluated in PTZ induced seizure tests, and maximal electroshock testing. The results showed that ZMEO is an efficient anticonvulsant, increasing latency to onset of tonic convulsion induced by PTZ, and by electroshock with effective doses (ED_50_) of 0.26 and 0.27 mL/kg respectively. Potential signs of neurotoxicity assessed in the Rota-rod test were found at 0.65 mL/kg. However, the lethal dose of ZMEO for 50% of the treated animals (LD_50_ = 2.35 mL/kg) was nine times greater than the effective dose. This suggests acceptable therapeutic effect for ZMEO [109]. Linalool, a major component of ZMEO, may contribute to this result, since it also presents anticonvulsive activity, however, more studies are needed to better characterize these interactions and effects [84].

### 2.14. Rosmarinus officinalis *L.*, Ocimum basilicum *L.*, Mentha pulegium *L.*, M. spicata *L.*, M. piperita *L.*, Origanum dictamnus *L.* and Lavandula angustifolia *Mill.*

**Figure 2:**
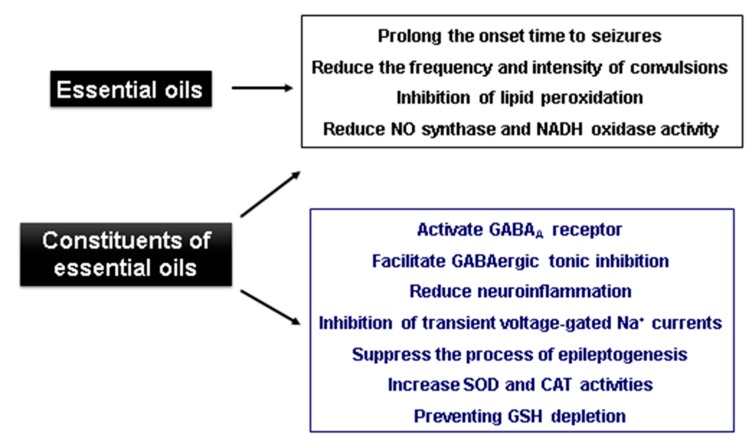
Mechanisms of anticonvulsant action of essential oils and their constituents.

The EOs of *Rosmarinus officinalis*, *Ocimum basilicum*, *Mentha pulegium*, *M. spicata*, *M. piperita*, *Origanum dictamnus* and *Lavandula angustifolia* extracted from Greek aromatic plants and administered at doses of 1.6 mL/kg i.p. in mice caused motor alterations, and in a few cases, lethargy. Evaluating anticonvulsive activity using the PTZ test, all of the oils tested promote seizure latency and a decrease in seizure intensity as compared to the control groups. The best results [110] were observed for *Mentha piperita*, whose EOs presented no convulsions in the treated animals. Figure 2 summarizes the main effects of essential oils.

## 3. Chemical Constituents

### 3.1. Alpha-Asarone

α-Asarone is a bioactive compound found in several plant species of the family Araceae (Acorus), and trees of the family Annonaceae, it is known for its neurological properties and effects on the CNS [111]. The compound is also known in the scientific literature for its extensive neuroprotective performance. According to Shin et al. [112], through reduction of proinflammatory cytokines and microglial activation in the hippocampus, α-asarone improves memory deficit induced by administration of inflammatory agents. Corroborating these results, Jo et al. [113] demonstrated that α-asarone reduces inflammation associated with injury to the spinal cord through attenuation of neuronal damage and promotion of angiogenesis. The anxiolytic effect of α-asarone has also been evaluated in an animal model of chronic pain, where α-asarone was effective in inhibiting anxiety by regulating neurotransmission and neuronal excitability [114].

Up to a dose of 1000 mg/kg, oral α-asarone caused no animal deaths, indicating a greater value than 1000 mg/kg for its LD_50_. Chronic (four-week) twice-daily treatments with α-asarone did not induce significant behavioral changes. The highest dose administered (200 mg/kg), caused a discreet sedative effect, and decreased spontaneous movements of the treated animals. The toxicity profile of α-asarone was analyzed by Chen et al. [115] who compared the efficacy of acute and chronic of α-asarone treatments in seizure prevention.

The researchers demonstrated that α-asarone presents moderate anticonvulsant activity when administered acutely, but the effect was potentiated when using chronic treatments, since at doses of 50, 100, and 200 mg/kg (p.o.) decreases were observed in tonic convulsion during the MES respectively in 40%, 20% and 0 in treated animals. These same doses reduced the incidence, increased latency, and decreased the duration of PTZ-induced seizures when compared to the negative control. In the model of epilepticus status induced by a combination of lithium and pilocarpine, chronic administration of α-asarone at doses of 100 and 200 mg/kg reduced the incidence of spontaneous seizures, seizure severity, and frequency of seizures during treatment (Table 2).

**Table 1:** Composition of plant essential oils and description of their anticonvulsant activity in nonclinical models.

Species	Essential Oil	Major Components Reported in the Literature	Major Components of the Evaluated Essential Oil	Experimental Protocol	Anticonvulsant Activity and/or Mechanism	Animal Tests and/or Cell Line Reference
*Bunium persicum* (Boiss). B. Fedtsch	Seed	γ-Terpinene (46.1%), cuminaldehyde (15.5%), *p*-cymene (6.7%) and limonene (5.9%) [31]	-	PTZ induced seizure test Maximal electroshock test	Prolonged onset time of clonic and tonic seizures [36]	Male NMRI mice
*Calamintha officinalis* Moench	Leaf	-	Carvone (38.7%), *neo*-dihydrocarveol (9.9%), dihydrocarveol acetate (7.6%), dihydrocarveol (6.9%), 1,8-cineole (6.4%), *cis*-carvyl acetate (6.1%) [40]	PTZ induced seizure test	Protected against generalized tonic- clonic seizures Decreased the number and duration of convulsions Reduced mortality [40]	Adult male Wistar rats
*Cinnamosma* *madagascariensis* Danguy	Leaf	Linalool (30.1%), limonene (12.0%), myrcene (8.9%) and α-pinene (8.4%) [44]	-	PTZ induced seizure test	Increased the latency period Reduced the frequency and intensity of convulsions [45]	Adult male and female Wistar rats
*Citrus aurantium* L. var. *amara*	Blossoms	-	Linalool (28.5%), linalyl acetate (19.6%), nerolidol (9.1%) and farnesol (9.1%) [53]	PTZ induced seizure test Maximal electroshock test	Produced protection against clonic Exhibited inhibition of the tonic convulsion [53]	Male NMRI mice
*Dennettia tripetala* G. Baker	Seed	β-Phenyl nitroethane (87.4%), linalool (8.8%) [116]	-	PTZ induced seizure test strychnine induced seizure test	Offered protection against PTZ- induced convulsion Flumazenil blocked anticonvulsant effect [58]	Adult male and female albino mice
*Elettaria cardamomum* L. Maton	Seed	-	1,8-Cineole (45.6%), α-terpinyl acetate (33.7%) [64]	PTZ induced seizure test Maximal electroshock test	Delayed onset of clonic seizures Increased onset time of tonic convulsions Reduced the percentage of hind limb tonic extension [64]	NMRI male mice
*Gardenia lucida* Roxb.	Apical buds and young shoots	*-*	α-Pinene (45%), spathulenol (31%) [67]	PTZ induced seizure test Maximal electroshock test	Protected against the intensity and frequency of convulsions, and mortality rate [67]	Male and female Swiss Albino mice
*Pimpinella anisum* L.	-	*-*	*trans*-Anethole (89.1%)	PTZ induced seizure test Electroencephalogram recordings	Prolonged time to appearance of seizures Decreased the frequency, amplitude, and duration of epileptiform burst discharges Showed neuroprotective effect [75]	Adult male Wistar rats
*Piper guineense* Schum & Thonn	Fruits	-	β-Sesquiphellandrene (20.9%), linalool (6.1%), limonene (5.8%), β-bisabolene (5.4%), α-pinene (5.3%) [82]	PTZ induced seizure test	Decreased mortality Reduced the Incidence of Convulsions [82]	Adult male and female albino mice
*Smyrnium cordifolium* Boiss	Plant	-	Curzerene (65.26%), δ-cadinene (14.39%), and γ-elemene (5.15%) [90]	PTZ induced seizure test	Prolonged onset time to seizure [90]	Mice
*Thymus vulgaris* L.	Fresh herb	-	Thymol (34.78%), *p*-cymene (14.18%), carvacrol (6.16%) and β-caryophyllene (5.46%) [94]	Maximal electroshock test	Protected against the convulsions	Male Swiss Albino mice
*Zataria* *multiflora* Boiss	-	-	-	PTZ induced seizure test Maximal electroshock test	Increased the onset time to clonic seizures Prevented tonic convulsions [102]	
*Zhumeria majdae* Rech	Aerial parts	-	-	PTZ induced seizure test Maximal electroshock test	Increased the onset time to tonic convulsions Prevented tonic Convulsions [109]	NMRI male mice
*Rosmarinus officinalis* L., *Ocimum basilicum* L., *Mentha pulegium* L., *M. spicata* L., *M. piperita* L., *Origanum dictamnus* L. and *Lavandula angustifolia* Mill.	-	-	-	PTZ induced seizure test	Increased seizure latency, decreased intensity, and differences in the quality of seizures, characteristics, from simple twitches to complete seizures [110]	Adult female white Balb-c mice

In cultures of hippocampal neurons, α-asarone suppressed the excitability of the cells via GABA_A_ receptor activation and tonic facilitation of GABAergic inhibition. This mechanism of action explains the anticonvulsant activity observed in kainate-induced seizure tests, since daily administration of α-asarone at a dose of 50 mg/kg (i.p.) for three days reduced the susceptibility of the mice to seizure [117]. α-Asarone (50–200 mg/kg, i.p.) was also effective in delaying the onset time of nicotine-induced clonic seizures, but did not prevent them from happening. These effects were not mediated through the antagonism of nicotinic acetylcholine receptors (nAChRs) [118]. Recently, Liu et al. [119] proved that α-asarone attenuates the memory and learning deficit caused by pilocarpine-induced status epilepticus in rats via suppression of proinflammatory cytokines, through decreased nuclear-*κ*B factor activation and reduced microglial neuroinflammation. 

In addition, administration of α-asarone (9 mg/kg, i.p.) revealed antioxidant activity in the hippocampus of rats when subjected to sound stress, through increased levels of SOD, CAT, GSH, vitamin C, vitamin E, and reduction of cerebral lipid peroxidation, thus attenuating memory deficit [120]. Moreover, α-asarone (15 μg/mL) exhibited neuroprotective activity in rat astrocyte cultures and was able to reduce ROS production and oxidative stress induced by *tert*-butyl hydroperoxide through attenuation of enzyme antioxidant depletion [121]. Thus, it is noted that the substance presents anticonvulsivant and neuroprotective activity.

### 3.2. Alpha- and Beta-Pinene

Alfa- and beta-pinene are two bicyclic monoterpenes with isomeric chemical structure found in the chemical composition of many essential oils [44,67,82]. Felipe et al. [122] evaluated the anticonvulsant effect of the two isolated terpenes as well as the racemic mixture. In the evaluation of the latency to the onset of the first seizure in the PTZ test, monoterpenes alone did not change this parameter, unlike that observed by the administration of the racemic mixture. Only beta-pinene and the mixture of the two monoterpenes, both at the dose of 400 mg/kg, p.o. were able increased the time of death of animals compared to the control group after PTZ-treated. Thus, it appears that only beta-pinene has anticonvulsant activity, however pre-treatment with α- and β-pinene and their equimolar mixture reduced concentration of norepinephrine, dopamine and nitrite concentration, but not TBARs concentration, on the striatal in relation to the PTZ-treated group.

### 3.3. (+)-ar-Turmerone

The main constituent of the aromatic plants *Curcuma longa* L. and *C. phaeocaulis* Valeton is sesquiterpene (+)-ar-turmerone which presents antitumor [123], larvicide [124], antifungal [125], and anti-inflammatory activity [126]. Liju et al. [127] found through in vitro tests that oil extracted from *Curcuma longa* L. rhizomes was able to sequester hydroxyl radicals (IC_50_ = 200 μg/mL), and superoxide anions (IC_50_ = 135 μg/mL), and inhibited lipid peroxidation (IC_50_ = 400 μg/mL). In addition, in vivo tests with mice were performed and based on the results, oral administration of 500 mg/kg increased levels of the antioxidant enzymes superoxide dismutase, glutathione reductase, and glutathione-S-transferase. The activity was mostly attributed to (+)-ar-turmerone, its principal metabolite (61.79%).

When assessing potential anticonvulsant effects of (+)-ar-turmerone (0.1–50 mg/kg), protective activity against convulsions induced by electrical and chemical (PTZ) stimulation was observed. The threshold needed to trigger seizures increased. Rapidly absorbed from the peritoneum to the brain, (+)-ar-turmerone can be detected after 15 min, and after 24 h from administration (i.p.), making it therapeutically promising. In zebra fish larvae, (+)-ar-turmerone was found to modulate the expression of two genes which are related to convulsion, c-fos, and brain-derived neurotropic factor (BDNF) [128].

### 3.4. βeta-Caryophyllene

β-Caryophyllene is a bicyclic sesquiterpene found in several EOs, such as *Aquilaria crassna* [129], *Croton campestres* [130], *Psidium guineense* [131] and *Zanthoxylum acanthopodium* [132]. β-Caryophyllene is the main component of *Cannabis* essential oil and is capable of binding to CB2 receptors. However, the compound can be classified as non-psychoactive cannabinoid [133]. Certain pharmacological properties have already been attributed to β-caryophyllene such as anticancer, anti-oxidant, and antimicrobial [129], as well as antidepressant [134] and anti-inflammatory [130]. In a pre-clinical study in female Swiss mice, no toxicological effects were observed up to a dose of 2000 mg/kg (p.o.) in animals treated with β-caryophyllene both acutely and repeatedly for 28 days [135].

In a study by Liu et al. [136], two-day pretreatment with β-caryophyllene (30 and 60 mg/kg, i.p.) reduced convulsant activity scores induced by intraperitoneal administration of kainate. When assessing the degree of kainate-induced neurodegeneration, β-caryophyllene was found to have a protective effect contributed to by increased activities of the antioxidant enzymes SOD, CAT, and GPx. The brain inflammation normally caused by kainate administration was reduced by β-caryophyllene, helped by inhibition of the proinflammatory cytokines IL-1β and TNF-α. In a study of Calleja et al. [137], the antioxidant activity of β-caryophyllene in rats (200 mg/kg, p.o.) was investigated. β-Caryophyllene was able to reduce lipid peroxidation as induced by carbon tetrachloride, demonstrating great ability to eliminate free radicals such as the hydroxyl radical and the superoxide anion. These results are consistent with results obtained by Dahham et al. [129], where the radical scavenging capability of β-caryophyllene was analyzed through the DPPH and FRAP methods with IC_50_ 1.25 ± 0.06 μM and 3.23 ± 0.07 μM, respectively. In addition, administration of β-caryophyllene in rats (50 mg/kg, i.p.) revealed neuroprotective activity, being able to inhibit lipid peroxidation, glutathione depletion, and promote an increase in levels of the antioxidant enzymes (SOD and CAT) in the brain, as well as reduce activation of astrocytes and microglial cells by attenuating neuroinflammation and ROS production in the central nervous system [138].

Recent studies have shown that β-caryophyllene (2.5 µM) reduced 1-methyl-4-phenyl-pyridinium-induced neurotoxicity in SH-SY5Y cells by reducing ROS production, increasing GSH levels and GPx activity, in addition to restoring mitochondrial membrane potential and inhibiting neuronal apoptosis [139]. Moreover, Askari and colleagues demonstrated that B-caryophyllene (0.5 and 1 µM) promotes neuroprotection through activation of type 2 cannabinoid receptors, promoting antioxidant action through nuclear factor-erythroid 2-related factor 2 (Nrf2) / HO-1 / anti-oxidant axis pathway [140], which promotes increased levels of GSH, CAT and SOD [141,142].

In the pentilenotetrazole-induced seizure model, β-caryophyllene (100 mg/kg, i.p.) increased the latency to myoclonic seizures as compared to the control group as confirmed using electroencephalogram, revealing that such changes also occur at electrophysiological levels. It is worth noting that β-caryophyllene at the dose in which it presented anticonvulsant activity improved the recognition rate for the object in the recognition test, without motor alterations in the open field, Rota-rod and forced-swim tests [143]. However, different from what was observed by Liu et al. [136], β-caryophyllene did not prevent oxidative stress as induced by pentylenetetrazole [136]. According to Tchekalarova et al. [144], β-caryophyllene (30, 100 and 300 mg/kg, i.p.) was effective on MES and PTZ tests when administered 0.5 and 4h before each experiment. In the kainate induced status epilepticus model, the pre-treatment with β-caryophyllene (50 and 100 mg/kg, i.p. for 7 days) alleviated the neurotoxicity and lipid peroxidation in the hippocampus of mice.

### 3.5. Borneol

Borneol is a bicyclic monoterpene widely used in the food industry, and in traditional Chinese medicine to treat pain and anxiety [145]. Borneol is found in many EOs such as *Lavandula angustifolia* Mill. [146], *Micromeria persica* Boiss. [147], *Perovskia abrotanoides* Kar. [148], *Thymus vulgaris* L. [149], *Rosmarinus officinalis* L. [150] and *Salvia officinalis* L. [151]. Recent studies have demonstrated the various effects of borneol in the treatment of chronic and neuropathic pain through activation of the GABAergic system and blocking transient receptor potential ankyrin 1 in the spinal cord [152,153]. Cao et al. [154] demonstrated that borneol may be a novel therapeutic agent for fear and anxiety disorders. Wu et al. [155] demonstrated that borneol increased blood-brain barrier permeability; related to increased ICAM-1 expression, thus becoming a promising candidate for treatment of brain tumors and infections, as well as chronic disorders in the central nervous system [155,156]. In an experimental model of permanent cerebral ischemia, borneol reduced the size of ischemia by decreasing expression of nitric oxide synthase and TNF-α in a dose-dependent manner [157].

In addition to these activities in the CNS, Tambe et al. [158] showed the antiepileptogenic effect of borneol. The authors proved that borneol (5, 10 and 25 mg/kg, i.p.) delays tonic seizures and progressive neuronal damage throughout the course of kindling. The oxidative stress triggered in the epileptogenesis model was drastically reduced by the administration of borneol at all doses tested, decreasing levels of lipid peroxidation which is directly related to production of free radicals and cell death. The activities of the antioxidant enzymes SOD, CAT, and GSH were elevated, and in addition, borneol also attenuates ROS production in neurons in the cerebral cortex of rats by reducing the expression and activation of inducible nitric oxide synthase (iNOS), as well as inhibiting neuronal apoptosis, thus promoting neuroprotection [159].

### 3.6. Carvacrol

Carvacrol is a phenolic monoterpene present mainly in the EOs of *Origanum vulgare* L., *Thymus vulgaris* L., *Lepidium flavum* Torr. and other aromatic plants [160,161]. Many studies have demonstrated its therapeutic activity which includes antimycobacterial [162], anticancer [163], antiasthmatic [164], antinociceptive [165], antifungal [166] and cardioprotective [167]. Carvacrol treatment has alleviated memory deficit in rats with Parkinson’s disease, but also promoted neuroprotection via non-specific blockade of TRPM7 receptors [168,169]. Corroborating these results, Celik and collaborators [170], investigating the neuroprotective effect of carvacrol in a methotrexate-induced toxicity model in rats found that the administration of carvacrol (73 mg/kg, i.p.) was able to increase the total antioxidant status level and significantly reduce levels of MDA, IL-1β, and TNF-α. In addition, administration of carvacrol (40 mg/kg, i.p) was able to reduce levels of MDA, GSH, SOD, GPx, and CAT in the brain [171]. In addition, administration of carvacrol (50 mg/kg) was able to reduce propiconazole-induced DNA damage on brain, through antioxidant mechanisms by increasing CAT, GSH and GPx levels [172]. Thus, carvacrol should exerting neuroprotective activity through antioxidant and anti-inflammatory mechanisms.

Misha et al. (2014) [173] demonstrated the anticonvulsant potential of carvacrol in the 6 Hz (32 mA) model, with an ED_50_ value of 35.8 mg/kg. In the epilepticus status (ES) model, animals receiving three doses of carvacrol (75 mg/kg i.p.) in the first 24 h developed less ES (25%) than the negative control group (~ 86%), but this same effect was not observed in chronic epilepsy. Interestingly, carvacrol inhibited TRPM7 channels, reducing neuronal death in the CA1 and hilar regions, with consequent prevention of SE-induced memory deficit [174]. According to Sadegh and Sakhaie [175], carvacrol prevented the proconvulsant effect of LPS possibly through the inhibition of the hippocampal COX-2 increased activity and preventing the neuroinflammation.

### 3.7. Carvacryl Acetate

Carvacryl acetate, obtained from acetylation of the monoterpene carvacrol, presents pharmacological activities described as antischistosomal [176], antinociceptive [177] and anti-inflammatory via interaction with the TRPA1 receptor [178]. Like carvacrol, carvacryl acetate presents activity in the central nervous system, presenting anxiolytic-like properties probably mediated by the GABAergic system, but without acting on 5-HT1A receptors [179]. The antioxidant potential of carvacryl acetate was notable in both in vitro and in vivo tests, increasing glutathione levels, and improving CAT, SOD, and GPx enzyme activity in the hippocampus of mice, while reducing lipid peroxidation, nitrite, and hydroxyl radicals. The results suggest possible use of carvacryl acetate in the treatment and prevention of neurodegenerative diseases related to oxidative stress [180].

Carvacryl acetate has also been shown to present anticonvulsant activity at a dose of 100 mg/kg (i.p.) in pilocarpine, PTZ, and picrotoxin-induced convulsion tests, where it respectively reduced the percentage of seizures by 60%, 30%, and 50% [181]. These effects were reversed by administration of flumazenil, suggesting that the mechanism of action may involve the GABAergic system, and corroborating the results obtained by Pires et al. (2013) [179]. It is known that epilepsy is related to failures in the function of the enzymes Na^+^, K^+^-ATPase, and δ-aminolevulinic acid dehydratase (δ-ALA-D) [182]. Treatment with carvacryl acetate (100 mg/kg i.p.) improved the activity of these enzymes, and also increased levels of δ-aminobutyric acid (GABA) in the hippocampus after induction of seizures with chemical agents [181].

### 3.8. Curcumol

One of the main components of *Curcuma longa* oil is the sesquiterpene hemiacetal curcumol, which has multiple therapeutic effects including anticancer [183], anti-inflammatory [184] and antifungal [185] activities have been attributed to the substance. The anticonvulsant activity of curcumol was evaluated after three consecutive days of previous treatment (100 mg/kg, i.p.). Seizure induced by pentilenotetrazole and kainate was observed as suppressed by increased latency for tonic and clonic convulsion, as well as reduced seizure severity and susceptibility [186]. The results can be explained by activation of GABA_A_ receptors in a benzodiazepine-independent site in hippocampal neurons, causing suppression of network hyperactivity [186,187].

### 3.9. Curzerene

Curzerene is a sesquiterpene found in curcuma rhizomes, mainly of the traditional *Curcuma longa* species. Pre-clinical studies have shown the antitumor [188], pesticidal [189] and antifungal [185] effects of this substance. According to Abbasi et al. [90], curzerene was effective in the PTZ test with an ED_50_ value of 0.25 ± 0.09 mg/kg. At a dose of 0.4 mg/kg, curzerene increased the latency time to first seizure, and decreased seizure duration as compared to the control group. At the same dose in the treated groups, there was 100% protection or zero mortality. The effects of curzerene can be attributed to activation of the GABAergic and opioid systems.

### 3.10. Epoxy-Carvone

Epoxy-carvone (EC) is a monocyclic monoterpene that can be found in the essential plant oil of *Carum carvi* L., *Catasetum maculatum* Kunth., and *Kaempferia galangal* L., among others [190,191]. Epoxy-carvone (EC) presents antinociceptive, and anti-inflammatory activity [192], antiulcerogenic [193], and spasmolytic activity in guinea pig ileum [194]. Relevant results have also been demonstrated in the pharmacological tests of motor coordination and induced sleep time [195].

The EC presents stereogenic centers that allow generation of four stereoisomers; (+)-*cis*-EC, (-)-*cis*-EC, (+)-*trans*-EC, and (-)-*trans*-EC, suggesting a comparative study of the anticonvulsant activity of these substances. In the PTZ-induced seizure test, all stereoisomers tested (300 mg/kg, i.p.) were effective in protecting against seizures, increasing latency for PTZ seizures with no deaths observed, and the (+)-*trans*-EC and (-)-*trans*-EC isomers respectively presented 25% and 12.5% inhibition of seizures. In the pilocarpine-induced seizure test, (+)-*cis*-EC, (+)-*trans*-EC and (-)-*trans*-EC were more effective in reduction of parameters related to cholinergic receptor activation, and increasing latency to the seizure onset. No significant results were obtained in the strychnine-induced seizure test for any of the isomers tested. In the model of maximal atrial electroshock, although they all significantly reduced seizure duration, there were differences in effect [196]. In the PTZ-induced kindling model with (+)-*cis*-EC and (-)-*cis*-EC, both reduce seizure severity and levels of proinflammatory cytokines IL-6 and TNF-α in mice hippocampi. The pre-treatment with the stereoisomer (+)-*cis*-EC showed neuronal protection in the epileptogenic process [197].

### 3.11. Eugenol

Eugenol is a natural and pharmacologically active aromatic substance present in plant EOs such as *Eugenia caryophyllus* Spreng., *Myristica fragrans* Houtt. and *Ocimum gratissimum* L. [198]. Also known as eugenic or cariophilic acid, eugenol is a phenylpropanoid; biologically synthesized from the amino acid phenylalanine through the shikimic acid metabolic pathway [199]. Eugenol has been widely used as an analgesic in dentistry due to its anti-inflammatory activity observed in cells of the dental pulp [200]. Several biological activities; hypoglycemic [201], platelet aggregation inhibition [202], antimicrobial [203], and antiulcerogenic [204] have all been reported for eugenol.

Jeong et al. [205] evaluated the inhibitory effect of eugenol after unilateral injection of kainic acid in the hippocampus; there was significant delay in seizure onset. At 200 mg/kg i.p., eugenol decreased the appearance of granule cell dispersion (characteristic of temporal lobar epilepsy) by 52%. These results can be explained by mammalian target of rapamycin (mTOR) signaling pathway deactivation (activation is related to the development of epilepsy). According to Huang et al. [206], eugenol inhibits sodium current, affecting activation of the action potential, with consequent neuronal hyperexcitability modulation. Yet, Vatanparasta et al. [207] reported that both the neuronal inhibitory and excitatory effects of eugenol are concentration dependent, i.e., low concentrations have antiepileptic properties and high concentrations induce epileptiform activity. In the lithium-pilocarpine epilepsy model, eugenol (100 mg/kg, i.p.) reduced the severity of seizures and animal mortality. The number of neurons in the dentate gyrus and CA regions of the hippocampus was reduced by administration of this substance, which when administered alone curiously decreased neuronal survival. Neuroprotective effect was also observed due to an increase in the antioxidant marker glutathione peroxidase [208]. Corroborating these results, in an experimental model of neurotoxicity in rats, oral supplementation of the diet with eugenol (50 mg) increased the total antioxidant status level and reduced lipid peroxidation and neuronal apoptosis promoted by aluminum chloride [209]. Similarly, pretreatment of mice with eugenol (10.7 mg/kg) attenuated lipid peroxidation and protein oxidation, and increased levels of Gpx, SOD, CAT, and GST antioxidant enzymes [210].

### 3.12. Gamma-Decanolactone

Gama-decanolactone is a monoterpene that acts on the central nervous system as anticonvulsant and hypnotic [211]. Gama-decanolactone has neuroprotective effect against seizures induced by isoniazid and 4- aminopyridine, but not by picrotoxin. These results suggest a possible modulation of GABA pathways and potassium channels directly or indirectly [212]. In the pilocarpine-induced status epilepticus, gamma-decanolactone at a dose of 300 mg/kg increased the latency to the first clonic seizure, but not there was no statistical difference in the group who received a dose of 100 mg/ kg compared with the control group. In this seizure model, gamma-decanolactone (100 and 300 mg/kg) suppressed the production of reactive oxygen species and DNA damage, as well as increased the activity of antioxidant enzymes and increased NO levels in the cerebral cortex of mice [213]. 

### 3.13. Linalool

Linalool is an acyclic monoterpene found as a volatile major constituent in the EOs of various aromatic plants [214,215]. Due to lower expression of (-)-(3R)-linalool synthase, Magnard et al. [216] has demonstrated lesser amounts of this monoterpene in rose flowers. EOs of *Aniba rosaeodora* Ducke. (rosewood), *Aniba parviflora* Meisn. (Macacaporanga), and *Aeollanthus suaveolens* Mart. (Catinga-de-mulata) are rich in linalool, which in synergism with other constituents, possesses antidepressant activity without compromising spontaneous locomotion or memory retention in treated animals [217]. Studies have revealed the excellent results of linalool in the central nervous system, promoting phospholipid homeostasis in neurological recovery after ischemia [218], as well as improvements in memory loss and behavioral effects in REM sleep deprivation modelling [219]. Linalool decreases cognitive deficit in mice as induced by intrahippocampal administration of β-amyloid protein; which is responsible for the pathogenesis of Alzheimer’s disease.

Linalool has demonstrated a neuroprotective effect via reduction of apoptosis and oxidative stress [220]. In study investigating the antioxidant activity of linalool in the rat brain, it was found that treatment with linalool, promoting neuroprotection (12.5 mg/kg, i.p.) is able to reduce the progressive gait abnormalities promoted by acrylamide, oxidative stress, lipid peroxidation, and increased GSH levels [221].

In central neurons of the snail *Caucasotache atrolabiata*, linalool at low concentrations (0.1 mM) demonstrated anti-(PTZ-induced) epileptic activity (20 mM), which was likely mediated by Na^+^ inward current, and indirect potentiation of Ca^2+^ activated K^+^ currents. However, high concentrations of this monotorpene (0.4 mV), augment neuronal hyperexcitability which is dependent on Ca^2+^ inward current and activation of protein kinase C [47].

Linalool oxide (OXL) is a monoterpene formed from the natural oxidation of linalool or through other synthetic routes. Although OXL is also present in EOs, it is found in lesser amounts [222,223]. Inhaled OXL presents anxiolytic effects without altering motor coordination [224]. The intraperitoneal OXL LD_50_ was estimated at 721 mg/kg with a confidence limit of between 681 and 765 mg/kg body weight. Evaluating anticonvulsant activity, OXL (50, 100, and 150 mg/kg, i.p.) significantly increased the duration of tonic seizures in maximal electroshock tests. In the PTZ-induced seizure testing, a 150 mg/kg dose alone was able to increase latency to the first seizure. Similar to linalool, OXL (50, 100 and 150 mg/kg, i.p.) did not cause either muscle relaxation or motor coordination deficits at any of the doses tested [225].

### 3.14. Nerolidol

Nerolidol is an aliphatic sesquiterpene and is a major compound found in the EOs of *Canarium schweinfurthii* Engl. [226], *Fraxinus dimorpha* Coss & Durieu [227], *Lindera erythrocarpa* Makino [228], *Myrcia splendens* Sw. [229] and *Piper aduncum* L. [230]. Many of nerolidol’s pharmacological effects; anxiolytic [231], antimicrobial [232] anti-inflammatory [233] and regression of endometriosis [234] have already been described in the literature.

According to Kaur et al. [235], nerolidol (12.5, 25, and 50 mg/kg, i.p.), in a model of epileptogenesis induced by pentylenetetrazole, presented protective effects since it reduced seizure severity. These results may be explained by an observed decrease in oxidative stress and favorable neurochemical changes including increased levels of noradrenaline, dopamine, and serotonin in both the cortex and the hippocampus of the treated animals. Nerolidol also improved depression and memory loss in the PTZ-kindled animals (psychiatric comorbidities associated with epilepsy.

In addition, administration of nerolidol (50 mg/kg, i.p.) to rats reversed neuroinflammation and cerebral oxidative stress by increasing levels of the antioxidant enzymes SOD, CAT, GSH and decreasing lipid peroxidation and MDA levels, besides reducing glial cell activation and dopaminergic neuron loss [236]. Nogueira-Neto and colleagues (2013) [237] found that treatment with nerolidol (75 mg/kg, i.p.) decreased oxidative stress in the mouse hippocampus, resulting in increased SOD and CAT activity, as well as reducing levels of nitrite and lipid peroxidation. The results reveal the therapeutic potential of nerolidol to treat and prevent brain diseases associated with oxidative stress.

### 3.15. 1-Nitro-2-phenylethane

1-Nitro-2-phenylethane (NPH) is a volatile compound found in the EOs of *Aniba canelilla* and *Dennettia tripetala* [58], and was the first nitrocomponent isolated from plants [238,239]. Several pharmacological activities for NPH have been reported, including anti-inflammatory [240], antinociceptive [241], trypanocidal [242], cytoprotective [243], anxiolytic and hypnotic effects [58]. NPH also presents vasodilator activity via stimulation of soluble guanylil cyclase [244], and induces vagal-vagal bradycardia in normotensive rats [245]. NPH presents an LD_50_ of 490 mg/kg (i.p.) in mice, indicating moderate toxicity. Pretreatment with NPH, (50, 100, and 200 mg/kg, i.p.), protected against PTZ-induced seizures (100%), and reduced mortality of the animals when compared to the PTZ-only control group. This effect was blocked by pre-administration of flumazenil, suggesting GABAergic involvement. In the strychnine-induced convulsion test, NPH at doses of 50, 100, and 200 mg/kg (i.p.) was effective in protecting against seizures by 20%, 80% and 100%, respectively, and provided protection against mortality, signaling possible glicinergic involvement [58].

### 3.16. Terpinen-4-ol

Terpinen-4-ol (4TRP) is a monoterpene found in the EOs of *Zingiber purpureum* Roscoe [246], *Artemisia caerulescens* L. [247] and *Hedychium gracile* Roxb [248], and *Melaleuca alternifolia* Cheel [249]. 4TRP presents several documented pharmacological effects that include antibacterial activity [250], anticancer [251], antifungal [252], antihypertensive [253], and anti-inflammatory activity [254], and is also widely used in the food, sanitary, and cosmetic industries [255]. Thus, Nóbrega et al. [256] performed studies demonstrating that although 4TRP modulates the GABAergic system, its activity is not reversed by pre-treatment with flumazenil, suggesting that the substance does not bind to the benzodiazepine site at the GABA_A_ receptor. When examining electroencephalogram changes caused by intra-cerebroventricular administration of 4TRP (100 and 200 ng/2μL) after PTZ injection, and compared to the control group there was an increase in latency for both myoclonic and tonic-clonic seizures. Surprisingly, 4TRP reduced Na^+^ and K^+^ currents in a concentration-dependent manner in dorsal root ganglion neurons, probably the main inhibition mechanisms for neuronal excitability and convulsive processes [256,257].

### 3.17. Thymol

Thymol is a phenolic monoterpene found mainly in EOs from the genus *Thymus* L. of Lamiaceae, and is an isomer of the monoterpene carvacrol. This compound is also found in the species *Lippia sidoides* Cham. [258], *Satureja macrostema* Moc. & Sessé ex Benth [259] and *Ocimum gratissimum* L. [260]. This bioactive compound presents a wide variety of pharmacological properties such as anti-obesity [261], anthelmintic [262], anti-inflammatory [263], hepatoprotective [264] and larvicidal [265].

Thymol has demonstrated antioxidant effects in mouse neuron cultures (10, 25, and 50 mg/L) [266], and presents excellent hydroxyl radical elimination capacity [267]. Thymol increases antioxidant enzyme levels, for example SOD, GPx, CAT, and GST, and non-enzymatic antioxidants such as vitamins C and E [268]. It has also been suggested that thymol (500 μM) presents a neuroprotective effect against oxidative stress induced by H_2_O_2_ in cortical rat neurons [269]. In addition, the administration of thymol (30 mg/kg) significantly reversed the amyloid-β-induced neurotoxic effects on the hippocampus of rats by increased GSH levels, reduced lipid peroxidation and decreased serum MDA levels [270]. Thymol also presents anesthetic and sedative activity in silver catfish via interaction with GABA_A_ receptors, but not at the GABA_A_/benzodiazepine site [271]. Thymol presented partial efficacy in MES, Metrazol (scMET), and Corneal-kindled models [173]. In the pentylenetetrazole-induced kindling model, thymol (25 mg/kg, i.p.) reduced seizure scores and malondialdehyde levels, as well as increased levels of glutathione. This antiepileptogenic effect can also be attributed to blocking Na^+^ channels post GABA_A_ receptor modulation. Locomotor capacity was also significantly reduced with the administration of thymol (25, 50, and 100 mg/kg, i.p.) [272].

### 3.18. (-)-Verbenone

Verbenone is a bicyclic monoterpene containing a ketone group commonly found in bark beetle pheromones. α-Pinene is a biosynthetic precursor [273]. Verbenone is produced by gut bacteria found in the red beetle *Dendroctonus valens*, and in medicinal plants such as *Verbena triphvlla* and *Eucalyptus globulus* Labill [274,275,276]. Verbenone presents good repellent properties, as well as anti-inflammatory [277] and bronchodilator [278] activity. Verbenone derivatives present important biological characteristics such as antifungal [279], antiviral [280], antioxidant activities and (in cortical neurons) anti-ischemic properties [281]. Given these promising effects, Melo et al. [282] evaluated the anticonvulsant activity of verbenone at doses of 150, 200, and 250 mg/kg (i.p.). Initially, they found that verbenone was unable to alter motor coordination in the animals and had no effect in pilocarpine-induced seizure test, as it did not alter latency to seizures or reduce peripheral cholinergic signals. Yet in the pentylenetetrazole-induced seizures test, verbenone (200 and 250 mg/kg) significantly increased the latency to onset of first seizure and reduced the percentage of tonic-clonic seizures, as well as the percentage of deaths when compared to the control group. Although the GABAergic system may be involved in this pharmacological activity, the effects were not reversed by flumazenil administration, showing that verbenone does not act at the same benzodiazepine binding site. Expression of BDNF and COX-2 in the hippocampus of animals receiving verbenone and PTZ were significantly increased, whereas c-fos was decreased. These results may be related to the neuroprotective effect of verbenone [283,284].

**Table 2:** Chemical structure and description of anticonvulsant activity in nonclinical models of essential oil constituents.

Compounds	Experimental Protocol	Anticonvulsant Activity and/or Mechanism	Animal Tests and/or Cell Line Reference	Reference
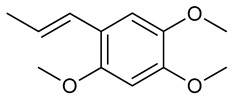 alpha-asarone	PTZ induced seizure test Maximal electroshock test Pilocarpine-induced seizures test	Decreased the occurrence of tonic hind limb extension. Reduced the hind limb extensor phase of convulsion Increased latency to seizure	Male Swiss mice and male Wistar rats	[116]
	Electrophysiological recording PTZ- and Kainate- induced seizure test	Enhanced tonic GABAergic inhibition Prolonged latency to clonic and tonic seizures	Rat hippocampal neurons and Male C57BL-6 mice	[117]
	Pilocarpine-induced *status epilepticus* rat model	Reduced learning and memory deficit Attenuated brain inflammation by inhibiting the NF-*κ*B activation pathway in microglia	Adult male Sprague-Dawley rats Microglia cell culture	[119]
	Nicotine-induced seizure test	Prolonged onset time to seizure, but not prevented the occurrence Did not interact with nicotinic acetylcholine receptors	Male ICR mice	[118]
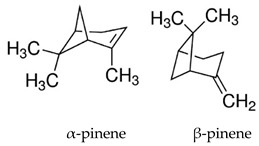	PTZ induced seizure test Neurochemical tests	Decreased the seizure intensity Reduced hippocampal nitrite level and striatal content of dopamine and norepinephrine	Male Swiss albino mice	[122]
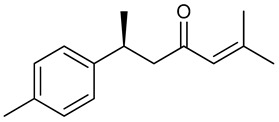 (+)-ar-Turmerone	6-Hz psychomotor seizure mouse model PTZ infusion model	Displayed anticonvulsant properties Modulated the expression patterns of seizure-related genes	Male C57BI/6 mice Male NMRI mice AB adult zebra fish	[128]
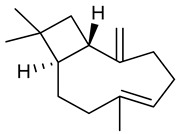 β-Caryophyllene	Kainic acid induced seizure test	Decreased the seizure intensity Reduced oxidative stress Reduced expression of TNF-α and IL-1β	Mice	[136]
	PTZ induced seizure test	Increased latency to myoclonic jerks	Adult C57BL/6 mice of both genders	[143]
	Maximal electroshock test PTZ induced seizure test Kainate induced status epilepticus	Suppressed tonic-clonic seizures Decreased seizure scores Decreased lipid peroxidation	Male albino ICR mice	[144]
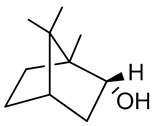 Borneol	PTZ-induced kindling model	Suppressed the process of epileptogenesis Reduced oxidative stress Prevented neuronal damage	Male Swiss albino mice	[158]
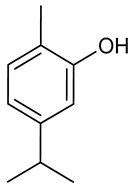 Carvacrol	6Hz psychomotor seizure test Maximal electroshock test PTZ induced seizure test Corneal kindling model Lithium-pilocarpine model	Prevented seizures in some tests.	Adult male CF No 1 albino mice	[173]
	Induction of SE Electrophysiological recording Immunohistochemistry Rewarded alternating T-maze test	Prevented memory deficits following SE Inhibited TRPM7 channels	Male adult Sprague-Dawley rats	[174]
	Lipopolysaccharide-PTZ induced seizure test	Prevented the proconvulsant effect of LPS Increased hippocampal level COX-2 but not COX-1	Adult male wistar rats	[175]
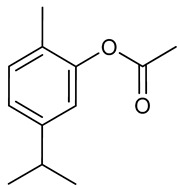 Carvacryl acetate	Pilocarpine- PTZ- Picrotoxin- induced seizure test Determination of Na^+^, K^+^-ATPase activity Determination of d-ALA-D activity Evaluation of amino acids levels in mice hippocampus	Increased latency to first seizure Reduced percentage of seizures Improved Na^+,^ K^+^-ATPase and d-aminolevulinic acid dehydratase activities Increased GABA levels	Male Swiss albino mice	[181]
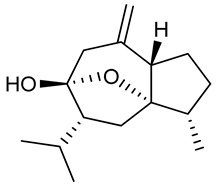 Curcumol	Electrophysiological recording PTZ- and Kainate- induced seizure test	Suppressed epileptic activity Facilitated GABAergic inhibition	Male C57BL/6J mice	[186]
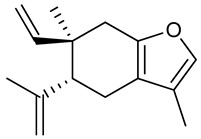 Curzerene	PTZ induced seizure test	Prolonged onset time to seizure and decreased the duration of seizure Effects on GABAergic and opioid systems	Mice	[90]
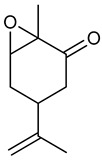 Epoxy-carvone	PTZ induced seizure test Maximal electroshock test Pilocarpine induced seizure test Strychnine Induced Seizure Test	Increased latency to seizure onset Prevented tonic seizures	Male Swiss albino mice	[196]
	PTZ-induced kindling model	Decreased seizure scores Decreased proinflammatory Cytokines Showed neural protection	Male Swiss albino mice	[197]
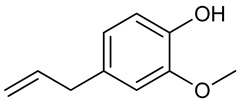 Eugenol	Intrahippocampal injection of kainic acid	Increased seizure threshold Reduced granule cell dispersion Suppressed mTORC1 hippocampal activation	Male C57BL/6 mice	[205]
	Electrophysiological measurements Pilocarpine-induced epileptic seizures	Inhibited transient voltage-gated Na^+^ currents Reduced percentage of severe seizures	Neuronal cells (NG108-15) Adult Sprague–Dawley (SD) male rats	[206]
	Intracellular recording	Induced inhibitory and excitatory effects in a concentration-dependent manner	Neurons of land snails *Caucasotachea atrolabiata*	[207]
	Lithium-pilocarpine model	Decreased seizure stages Reversed oxidative stress Increased cell survival in hippocampal sub-regions	Male rats	[208]
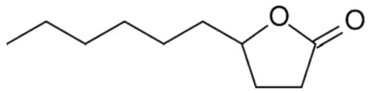 γ-Decanolactone	Isoniazid-, picrotoxin- and 4- aminopyridine- Induced Seizure Test	Prolong the latency to the first seizure Decreased the percentage of seizures	Male CF1 mice	[212]
	Pilocarpine-Induced Seizure Test	Prolonged the latency to first clonic seizure and reduced oxidative stress	Male CF1 mice	[213]
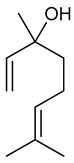 Linalool	PTZ induced seizure test	Suppressed action potentials at lower concentration. Excitatory effect in higher concentration.	Central neurons of snail *Caucasotachea atrolabiata*	[47]
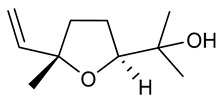 Linalool oxide	PTZ induced seizure test Maximal electroshock test	Increased latency to first seizure onset Reduced the duration of tonic seizures	Male Swiss albino mice	[225]
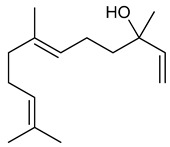 Nerolidol	PTZ-induced kindling test	Increased NE, DA, 5-HT in cortex and hippocampus Reduced oxidative stress	Male lake mice	[235]
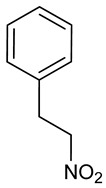 1-Nitro-2-phenylethane	PTZ induced seizure test strychnine induced seizure test	Offered protection against PTZ- strychnine-induced convulsion Flumazenil blocked anticonvulsant effect	Adult male and female albino mice	[58]
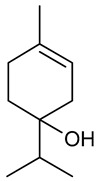 Terpinen-4-ol	PTZ induced seizure test 3-MP test Electroencephalogram recordings Dissociation and Patch-Clamp Recordings	Increased the latency to seizures Reduced the total time spent in generalized convulsions Reduced Na^+^ currents in a concentration-dependent manner	Adult male Swiss mice	[256]
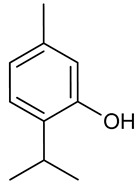 Thymol	6 Hz psychomotor seizure test Maximal electroshock test PTZ induced seizure test Corneal kindling model Lithium-pilocarpine model	Prevented seizures in some tests.	Adult male CF No 1 albino mice	[173]
	Maximal electroshock test PTZ-induced seizure test Strychnine-induced seizure test 4-Aminopyridine seizure test PTZ-induced kindling test	Reduced seizure scores Could block Na^+^ channels post GABA_A_ receptor modulation	Male Wistar rats Male Swiss albino mice	[272]
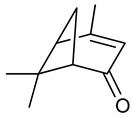 (-)-Verbenone	PTZ induced seizure test	Increased latency to onset of first seizure Reduced the percentage of tonic-clonic seizures Up regulated mRNA expression of BDNF and COX-2 Down regulated mRNA expression of c-fos	Male Swiss mice	[282]

3-MP: Mercaptopropionic Acid.

## 4. Discussion and Future Perspectives

Aromatic plants produce lipophilic volatile compounds able of crossing the blood-brain barrier and modulating neuronal changes involved in seizure. EOs extracted from these plants are rich in lipophilic secondary metabolites with great chemical complexity and can interact in several biological targets simultaneously. For example, *Cinnamosma madagascariensis* oil has anticonvulsant activity, probably due to the effects of its major constituents (linalool, limonene and myrcene) on the central nervous system [46]. Curzerene and linalool also have anticonvulsant activity and are the major components of *Smyrnium cordifolium* and *Zhumeria majdae* oils, respectively, which also have anticonvulsant effect [90,109]. However, some active principles have not yet clarified their biological activity, limiting the understanding of the effects observed in essential oils. Apiaceae and Lamiaceae families were the most promising because they had more essential oils with anticonvulsant activity. Among the bioactive compounds, β-caryophyllene, borneol, eugenol and nerolidol were the only ones that presented antioxidant properties related to anticonvulsant action reported in the literature. In this review, it was observed that essential oils and their constituents exert pharmacological action mainly via modulation of GABAergic neurotransmission. For example, *Dennettia tripetala* oil together with its constituent, 1-nitro-2-phenylethane, interact with the benzodiazepine receptor, as well as with curzerene [58,90]. α-Asarone reduces neuronal excitability via enhancing GABA tonic inhibition in vivo [117]. Carvacryl acetate increases GABA neurotransmitter levels in the hippo-campus [181]. Another important mechanism of EOs compounds is the reduction of seizure-induced inflammation and/or oxidative stress as observed in α-asarone, (-)-verbenone, β-caryophyllene, borneol, carvacryl acetate, eugenol and nerolidol [117,136,158,181,208,235,282]. Some constituents of EOs such as eugenol, terpinen-4-ol and thymol are able to reduce seizures via the modulation of ion channels [206,256,272]. It is worth noting that the constituents of EOs differ in chemical structure and in the position of their functional groups. According to Sousa et al. [285], the stereogenic center at the C-3 atom and the presence of a double bond influences the pharmacological potency of monoterpenes. Enantiomers of the same compound may have different biological activity or different potencies. For example, (*S*)-(+)-carvone was effective in protecting animals from seizures induced by chemical stimulation, while (*R*)-(+)-carvone was not effective [41]. Although this review emphasizes the beneficial effects of essential oils on seizure treatment, it is necessary to demystify the belief that natural products are not toxic [286]. Mathew et al. [287] reported ten cases of inhalation-induced seizures caused by eucalyptus oil, that is widely used for pharmaceutical purposes. Researchers believe that the epileptogenic effect of eucalyptus oil is due to cellular hyperexcitability [288]. Camphor EO and its major constituent, 1,8-cineole, caused signs of neurotoxicity such as seizures in rats [289]. In this sense, the need for further studies on the safety profile and mechanism of action of EOs is evident.

## 5. Materials and Methods 

The present study was based on a literature review of plants essential oils and their isolated constituents with anticonvulsant activity in animal models and antioxidant action. The search, performed in the Pubmed database, from January 2011 through December 2018 used the following keywords: anticonvulsant and seizure, essential oils, monoterpene and antioxidant.

## 6. Conclusions

Evidence on the role of oxidative stress in the biochemical and neurological events of epilepsy has been established. The studies discussed show that essential oils and their constituents with anticonvulsant activity can inhibit these processes via antioxidant action, in addition to other mechanisms of action that these natural products present on neuronal excitability. The inhibition of seizures exerted by the essential oils in various animal models, using chemical and physical convulsivant agents, demonstrate their therapeutic potential against various neurological disorders, especially epilepsy. Investigating the metabolization of these bioactive products, their subchronic and chronic toxicities and the consequent clinical screening are important steps to advance in obtaining phytochemicals as candidates for new antiepileptic drugs.

## Abbreviations

4TRP	Terpinen-4-ol
δ-ALA-D	δ-aminolevulinic acid dehydratase
ATP	Adenosine triphosphate
BDNF	Brain-derived neurotropic factor
BPEO	*Bunium persicum* essential oil
CAEO	*Citrus aurantium* essential oil
CAT	Catalase
CMEO	*Cinnamosma madagascariensis* essential oil
CNS	Central Nervous System
COEO	*Calamintha officinalis* essential oil
DTEO	*Dennettia tripetala* essential oil
DPPH	2,2-diphenyl-1-picrylhydrazyl
EC	Epoxy-carvone
ECEO	*Elettaria cardamomum* essential oil
EOs	Essential oils
ES	Epilepticus status
FRAP	Ferric reducing antioxidant power
GABA	δ-aminobutyric acid
GC/MS	Gas chromatography coupled to mass spectrometry
GLEO	*Gardenia lucida* essential oil
GSH	Glutathione reductase
GST	Glutathione S-transferase
GPx	Glutathione peroxidase
IL-1β	Interleukin 1β
iNOS	Inducible nitric oxide synthase
MDA	Malondialdehyde
MÊS	Maximal electroshock seizure
nAChRs	Nicotinic acetylcholine receptors
mTOR	mammalian target of rapamycin
NPH	1-Nitro-2-phenylethane
OXL	Linalool oxide
PAEO	*Pimpinella anisum* essential oil
PGEO	*Piper guineense* essential oil
PTZ	Pentylenetetrazol
RNS	Reactive nitrogen species
ROS	Reactive oxygenated species
scMET	Metrazol
SE	Epilepticus status
SCEO	*Smyrnium cordifolium* essential oil
SOD	Superoxide dismutase
TNF-α	Tumor necrosis factor α
ZHMEO	*Zhumeria majdae* essential oil
ZMEO	*Zataria multiflora* essential oil
